# Supplementary material for: Are carbon emissions trading and green financial instruments synergistic? -Comprehensive quantitative research based on content analysis
Source: PLoS One. 2024 Mar 7;19(3):e0298601. doi: 10.1371/journal.pone.0298601 (PMC10919601; doi:10.1371/journal.pone.0298601)
Supplement: S1 Data — (ZIP) [file pone.0298601.s001.zip › Data/地级市Z_F工作报告.zip!/╜¡╦╒/╒≥╜¡╩╨/└┤╫╘╣1⁄2╓┌║┼í╛┬φ┐╦╩2╛▌═°í┐_2004.docx]

各位代表：

　　现在，我代表镇江市人民政府，向大会作工作报告，请予审议，并请市政协各位委员和其他列席人员提出意见。

　　2003年工作回顾

　　2003年是新一届政府施政的开局之年，是镇江"立志两率先、奋力两步走，争当苏南后起之秀"的起步之年。全市人民在市委的领导下，深入贯彻十六大精神，认真实践"三个代表"重要思想，抓住机遇发展，迎着挑战奋进，经济社会发展取得新成绩，较好地完成了市五届人大一次会议确定的各项任务。

　　（一）国民经济健康快速发展，综合实力进一步增强。全市实现国内生产总值643.6亿元（预计数，下同），比上年增长14.1％。财政总收入73.9亿元，增长30.1％，占国内生产总值的比重达11.5％，比上年提高1.4个百分点。全社会固定资产投资237.1亿元，增长27.8％。第一产业实现增加值32.6亿元，增长4.1％。农业结构调整步伐加快，产业化经营稳步推进，优质粮油、高效园艺、特种水产、优质肉奶、生态经济林业、观光休闲农业等六大特色产业竞争力进一步增强。外资、民资、工商资本投入农业达20.5亿元，增长62.9%。获得无公害、绿色食品和有机食品认证的农产品品牌累计已达66个，新增30个。市区建立农产品市场安全准入制度。以水利为重点的农业基础设施建设得到加强，农业综合开发、土地复垦、农机化发展目标如期完成，农村现代化建设、茅山老区扶贫开发取得新的进展。第二产业实现增加值366.9亿元，增长16.2％。工业经济高开高走，速效同增，超额完成全年任务。规模以上工业企业完成增加值227.5亿元，增长22.5%；实现销售收入762.3亿元、利税62.1亿元，分别增长23.1％和31.3％。完成工业性投入120亿元，增长32.3%。128个省、市重点项目竣工投产，索普二期、大亚木业、鼎胜铝业、精功汽车等一批在建重大项目进展顺利。销售超亿元的企业110家，新增21家，规模支撑效应进一步放大。光电子及通信元器件产业基地和电力电器产业基地被评为全国先进火炬计划特色产业基地，沿江绿色化工产业基地通过科技部组织的专家评审。高新技术产业销售收入和实现利税分别增长31.5％和33.1％。辖市（区）工业经济运行质量明显提高，区域发展活力进一步增强。第三产业实现增加值244亿元，增长12.4%。社会消费品零售总额168.2亿元，增长12.6%。旅游业总收入63.3亿元，增长19.2%。焦山、宝华山建成国家4A级风景区。非典之后，采取针对性措施，促进服务业快速复苏，商品房、私家车、信息服务业等新的消费热点持续升温，社区服务业蓬勃发展。金融机构积极拓展资金渠道，优化信贷结构，加大对重点企业、私营个体经济和基础设施建设的支持，年末存款余额580.9亿元，贷款余额425.1亿元，分别增长27％和29.3％。

　　（二）新一轮沿江开发全面实施，对外开放跃上新平台。制订《沿江产业带规划》及其实施意见，市区及各辖市沿江开发全面推进。镇江新区开发框架迅速拉开，出口加工区通过国家验收并开始封关运作；丹阳开发区加速向滨江拓展；句容利用外资增势强劲；扬中开发区基础设施建设步伐加快；丹徒生态工业园项目落户势头迅猛；京口、润州沿江开发水平明显提升。5个省级开发区完成基础设施建设投入18.7亿元，比上年增长2.5倍。沿江新批投资1000万美元以上项目85个。润扬大桥及南接线工程建设加快，长江镇扬河段二期整治工程顺利完成，沪宁高速扩建、312国道拓宽、镇溧高速和宁常高速新建等项目相继开工。成功组织2003金秋经贸洽谈会及多场境内外招商活动。全年合同利用外资17.9亿美元，实际利用外资8.06亿美元，分别增长74.4%和60.8%。外贸、外经工作进一步拓展，实现进出口总额26.1亿美元，其中出口12.15亿美元，增长20.2%。投资环境进一步优化，发放外来投资企业"一卡通"，规范行政事业性收费，对影响环境的行为实行责任追究。海关、商检等六部门网上并联操作的口岸电子政务中心开始运行，建成可视远程监控系统。完成外贸运量620万吨，集装箱13.5万标箱，港口货物吞吐量3609万吨。

　　（三）各项改革纵深推进，机制活力明显增强。国企改革稳步推进，共改制改组国有企业152家。市直中小型工业企业和商贸、建设、外经贸企业改制目标基本完成，江奎、焦化两家大中型骨干企业改制取得突破，改制后企业发展势头良好。嘉新京阳水泥在香港主板市场成功上市。民营经济发展迅猛，吸纳民资达115.6亿元，新增注册资本73.8亿元，增长121.5％，新增私营企业3132户、个体工商户1.58万户。私个经济完成增加值157.7亿元，增长24.7%，占GDP总量的24.5%，比上年提升2.1个百分点。市属事业单位转企改制试点进展顺利。完成第二轮行政审批制度改革，成立市行政服务中心。完成港口管理体制改革。农村税费改革进一步深化，农民负担比上年下降4%。土地入市交易23亿元，比上年净增14亿元，土地市场化运作的成功实践，有力推动了国企改革、城市建设和社会发展。

　　（四）城市建设步伐加快，"三年大变样"目标顺利实现。完成《城市总体规划（2002-2020）》修编，重点工程规划水平明显提高。全年城建总投资101亿元，城市框架进一步拉开，城市功能日益完善。新建和改造檀山路、跑马山路、谷阳西环路、学府路、禹山北路、润州路、黄山南路、东吴路、梦溪路等城区主次干道21条，总长40.6公里。整治出新了丁卯桥路、朱方路、桃花坞路和东吴路北段。建成解放路南端高架、垃圾填埋场和污水处理厂。铁路三线搬迁完成一期工程，建成上隍货场。博物馆新展厅、人防指挥中心、城市防洪工程建设顺利推进。完成街巷改造面积1.01万平方米，并有计划地保护和修复古建筑。新建商品房83万平方米。新增京口广场、润州广场、花山湾广场、山巷广场、丹徒新区广场、东吴路绿地等一批标志性绿地，人均公共绿地新增1平方米，群众居住条件和环境进一步改善。创建国家卫生城市获得成功，创建国家环保模范城市通过国家级考核验收，荣获省级园林城市和全国园林绿化先进城市称号。社区结对共建和资源整合成效明显，建成82个示范社区，占社区总数的51%。成立行政执法局，出台城管重心下移办法，市区54条主次干道实现20小时保洁。城市长效管理机制初步形成。城镇建设步伐加快，城乡面貌明显改善，城市化率提高到52.3%。

　　（五）人民生活质量进一步提高，各项社会事业全面进步。城镇居民人均可支配收入9451元，农民人均纯收入4718元，分别增长15.2%和6%。城乡居民储蓄余额达338.2亿元，比年初增加57亿元。加大就业和再就业工作力度，城镇就业人数净增2万人，城镇登记失业率3.88%，低于全省控制指标。农村劳动力转移新增4.3万人，其中"康桥工程"完成劳务输出1.78万人。社会保障体系不断完善。社会养老保险"扩面征缴"，全市比上年净增1万人，养老金社会化发放得到巩固，基金收支当年基本平衡。社会医疗保险参保人数净增4.8万人。全市城乡有5900余户、1.52万人享受最低生活保障。18件为民办实事项目全面实施，完成情况良好。在51个具体项目中，完成43项，占84.3％；跨年度的5项，占9.8%；需作调整的3项，占5.9％。（18件实事的实施，得到了广大市民的热情支持，在这里我代表市政府向全市人民表示感谢。同时，对施工中给群众的生活和出行带来的不便，也表示歉意，并注意在今后的工作中更好地统筹安排。）农村"五件实事"进展顺利，草危房改造1060户，农村改水新增受益人数22.7万人，农村公路建成328公里、在建161公里、完成投资1.44亿元，农村合作医疗保险参保108.2万人，覆盖率达66.7%。全市上下万众一心、抗击非典，特别是广大医务工作者作出了很大贡献，全市未发生一起非典病例。连续第四次荣获"全国双拥模范城"称号。退役士兵全面实行"双向选择"和货币化安置。市和各辖市（区）均顺利通过全国科技进步先进城市考核。全年共组织实施市以上各类科技项目1106项，科技进步对工业和农业贡献额分别达到48.5%和55%。"镇江水环境质量改善与生态修复技术及示范"项目被列入国家863重大科技专项。九年义务教育质量提高，初中毕业生升学率达95%，适龄人口接受高等教育的比例达43.4%。新创建6个教育现代化乡镇，中小学布局调整积极实施，地方高校发展步伐加快。创新人才政策体系，参与长三角人才开发一体化建设，引进具有本科以上学历或中级以上专业技术资格的人才2310人。成功承办全国乒乓球锦标赛、男子排球锦标赛和少年象棋赛，全民健身服务体系"八个一"工程全面推进。辖市（区）、乡镇光纤联网率均达100%。广电中心建设、数字电视工程等重点项目启动。计划生育争创示范工程扎实推进。首次派出市民团访问日韩友好城市。党校、行政学院新楼落成，市级机关事务管理工作保障有力。建成市老年活动中心。统计、审计、人武、史志、档案、侨台、民族宗教、新闻出版、防震减灾、气象等工作取得新成绩，妇女儿童、残疾人、关心下一代等事业有了新发展。

　　（六）精神文明活动深入开展，民主法制建设进一步加强。制定并实施《2003-2012年文化名市建设规划纲要》，广泛开展"新时期镇江精神"大讨论，群众性精神文明活动生机勃勃。与中央电视台合作，摄制完成大型历史文献片《独领风骚--诗人毛泽东》。《激情广场》等大型专场文艺演出和《欢乐家园》系列广场文化活动取得圆满成功。民间文化艺术馆建成开馆。开展创"学习型、服务型、勤廉型"机关活动，政府自身建设得到加强。扎实推进依法治市、依法行政。自觉接受人民代表大会及其常委会和人民政协监督，办理人大代表建议182件、政协提案449件。行政执法和行政复议水平不断提高，政务公开继续在全省保持领先水平。积极开展"建设平安镇江、创建最安全地区"活动，严厉打击各种犯罪活动，社会治安综合治理和平安创建工作在全省名列前茅。严格执行廉政责任制，狠抓反腐倡廉各项工作的落实。坚持并完善党政领导信访接待日制度，切实履行社会稳定领导责任制，建立健全信访督查制度，认真做好人民来信来访工作，积极疏导化解社会矛盾。

　　过去的一年，镇江发生了许多令人振奋的变化，镇江在苏南板块的形象进一步提升，城市面貌的巨变引起省内外的关注。全市上下加快发展的信心更足，率先发展的氛围更浓，建设家乡的热情更高。虽然遇到的困难比预想的多，但取得的成绩比预期的好。我们在工作中，着重把握了以下四条：一是立率先发展之志，兴创新实干之风。我们坚持跳出镇江看镇江、立足苏南看镇江、放眼长三角看镇江，通过与周边先进地区比，既比出差距、比出压力，更比出斗志、比出信心；通过压力的层层传递，使广大干部心中都有目标，肩上都有责任，拼搏奋进，抢位争先。在应对挑战中把握主动，在扬长补短中增创优势，在超越自我中寻求突破，实现了"两率先、两步走"的良好开局。二是以重点突破之策，取推动全局之功。我们坚持把沿江作为开放的第一品牌，以大投入、快开发、强招商??坚持把发展作为改革的第一取向，以算大账、算长远账、算全局账的眼光，积极引进战略投资者，致力于把企业做大做强；坚持把特色作为城建的第一追求，以高起点、高标准、大手笔的力度，拉开城市框架，提升城市品位，形成了各类投资者"抢滩镇江"的态势。三是尽执政为民之责，办富民利民之事。我们在指导思想上明确：发展是硬道理、富民也是硬道理，发展要率先、富民也要加速。采取一系列措施，增加收入，扩大就业，纾解民困，最大程度地凝聚民心，开启民智，激发民力。让人民群众在更快更多地享受到改革发展成果的同时，也更加关心镇江发展、支持家乡建设。四是抓改进政风之本，收优化环境之效。我们强调政府行为是最重要的投资环境，强调实现"两个率先"，政府必须理念率先、行为率先、效率率先。以此为方向，不断加强政府自身建设，努力增强政府的公信力、亲和力和感召力，初步塑造出现代政府的良好形象。

　　各位代表，上述成绩的取得，是党的基本理论、基本路线和基本经验指引的结果；是省委、省政府和市委正确领导，市人大、市政协大力支持的结果；也是全市人民团结奋斗、共同努力的结果。在此我代表市人民政府，向全市广大工人、农民、知识分子、干部和离退休老同志，向各民主党派、工商联、人民团体和社会各界人士，向驻镇解放军、武警官兵和公安干警，向所有关心支持镇江发展的海内外朋友，表示衷心的感谢并致以崇高的敬意！

　　在肯定成绩的同时，我们也清醒地看到，经济社会发展中还存在一些矛盾和问题，主要是：产业层次还不够高，规模企业支撑作用还不够明显；利用外资、民资的实际到位率偏低；农民增收困难，特别是种粮农民收入增长缓慢；城乡居民就业压力较大，社会不稳定因素还比较多；少数政府部门自我加压、开拓创新的意识还不够强，不规范行政行为依然存在，发展环境还有待进一步优化。这些矛盾和问题已引起我们高度重视，并将在今后的工作中认真加以解决，也恳请各位代表和委员对我们的工作给予更多的关心、支持和监督。

　　2004年主要工作

　　新的一年，我们面临着重要的战略机遇，加快发展的环境继续趋好。国际产业资本加速向我国转移，长三角地区的联动发展，全省沿江开发战略的积极实施，为我们在更高起点、更大平台上加快发展提供了新契机。进入新世纪以来的成功跨越，更为镇江实现率先发展目标打下了坚实的基础。我们将全面贯彻党的十六大和十六届三中全会精神，认真落实市委四届七次全体（扩大）会议的部署，按照"率先、奋进、富民、务实"的总体要求，坚持"创新提升、务实推进"的工作基调，以加快富民为核心、改革开放为动力、结构调整为主线，抢抓沿江开发的机遇，加快融入长三角的步伐，促进经济社会全面、协调、可持续发展。

　　今年全市国民经济和社会发展的预期调控目标为：国内生产总值增长13.5%。全社会固定资产投资284亿元，增长20%。全口径财政收入84.3亿元，增长14%。合同利用外资22.4亿美元，实际利用外资10.1亿美元，分别增长25%；进出口总额30亿美元，其中出口14亿美元，增长15%。社会消费品零售总额188.4亿元，增长12%。城镇居民人均可支配收入10585元，增长12%；农民人均纯收入5050元，增长7%。城镇登记失业率控制在4%以内。人口自然增长率控制在2‰以内。工作中我们将力争做得更好。

　　同时，着力办好与市民生活密切相关、群众迫切需要解决的15件实事：

　　1．新增城镇就业2.2万人，本市农村劳动力转移6万人；

　　2．完成农村"五件实事"年度目标，推进城乡共同发展；

　　3．提高征地补偿标准，进行被征地农民基本生活保障制度试点，保障农民合法权益；

　　4．建成主城区防洪墙，整治团结河，提高城市防洪排涝能力；

　　5．建成10万平方米经济适用房，廉租房补贴受益面扩大50%，进一步缓解市区特困户住房困难；

　　6．拆除沪宁铁路城区南货场至西货场老支线，畅通城市主干道；

　　7．市区新增60辆公交车，新开通和优化5-7条公交线路，方便市民出行；

　　8．改造市区街巷和危房各1万平方米，整治小圩子路、黎明河和孩儿河沿线等积水区，改善市民生活环境；

　　9．完善社区基础设施建设，加强社区服务和管理，市区示范社区达标率和安全小区创建率达到70％以上；

　　10．新建3个城区农贸市场，方便市民生活；

　　11．新增蔬菜生产基地2000亩，丰富市民"菜篮子"；

　　12．完成市一中新校区一期工程和镇江中学的扩建；

　　13．加强体育设施建设，完成市体育中心立项和规划设计工作；

　　14．建成正东路健康广场，开工建设市疾病预防控制中心和市传染病防治医院，实施特困职工医疗救助；

　　15．完成市广电中心一期土建工程，开通市区数字电视。

　　围绕上述目标任务，我们将扎实做好以下六方面的工作：

　　（一）突出富民优先，确保人民生活水平新提高

　　鼓励创业富民。认真抓好市委、市政府《关于加快提高城乡居民收入的意见》的落实。大力宣传创业政策和创业典型，在全社会形成"政府创造环境，人民创造财富"的氛围，兴起全民创业、自主创业热潮。鼓励科技人员离岗创业，下岗职工学技创业，村干部带头创业，广大农民外出创业。扶持退伍转业军人和大中专毕业生自主创业。降低创业门槛，建立创业基金，落实优惠政策。加快筹建市、县、乡三级创业指导中介服务机构，帮助群众兴办各种实业。

　　推进就业安民。坚持就业优先，充分发挥劳动密集型产业和中小企业吸纳劳动力的中坚作用，把发展商贸流通、餐饮娱乐、交通服务和旅游业作为扩大就业的主攻方向，把社区服务业作为大龄失业人员再就业的重要渠道。完善相关政策，鼓励多形式灵活就业。充实小额贷款担保基金，实施社保补贴和岗位补贴，强化"订单式"技能培训，购买公益性岗位，力争年内实现双失业、双下岗家庭至少有1人就业。逐步统一城乡劳动力市场，设立专项资金，完善市、县、乡三级职业培训体系，发展和规范劳务中介组织，扎实推进"康桥工程"，年内力争户均1人以上实现非农就业。

　　实施保障济民。加强社会保险基金的征缴和管理。将社会保险扩展到城镇各类从业人员。抓好农村养老保险制度试点。确保城镇居民最低生活保障"应保尽保"，扩大农村最低生活保障的覆盖面。规范企业用工行为，严厉查处拖欠职工工资特别是民工工资等违法行为。加大对弱势群体的援助帮扶，加强社会医疗救助和法律援助工作，继续做好茅山老区的扶贫开发。

　　（二）突出层次提升，实现经济发展新跨越

　　加快农村经济发展。把解决好"三农"问题作为全局工作的重中之重。优化农业结构，依靠科技进步发展现代农业。引导外资、民资和工商资本投向农业，力争达到26亿元，增长26.8%。通过专业化布局、标准化生产、产业化经营，发展精深加工，提高农业的产出效益和附加值。努力提高乡镇企业发展水平，吸纳农村劳动力新增2.1万人。依托城镇，兴办特色专业市场，发展农村第三产业，提高对农民增收的贡献份额。尊重农民意愿，推行土地入股，加速土地流转，使出让承包经营权的农民获得稳定、持久的收入。实行最严格的耕地保护措施，稳定和提高粮食综合生产能力。加强农村基础设施建设，增强抗洪排涝能力，加快农业机械化进程，努力改善农民的生产生活条件。

　　做优做强工业经济。坚持走新型工业化道路，加快产业结构优化升级，从根本上提高经济增长质量和效益。重点抓好106个销售3000万元以上新增长点，抓好100个年销售5000万元以上的重点产品。进一步提高工业??170亿元，增长41.7％。加快推进总投资177亿元的50个省、市重点技改项目。把高新技术产业作为引领工业发展的先导力量，提高"一园四基地"建设水平，重点培育八大高新技术产品群，新建1-2个省级工程技术研究中心，力争高新技术产品销售收入突破230亿元。进一步发展光电子产业，积极参与国家半导体照明工程建设。大力发展规模经济，集中财税、贴息、科技三项费用等各种调控手段，扶持重点骨干企业。引导面广量大的中小企业朝着"专、精、特、新、高"的方向发展，建立专业化协作配套体系，通过产业成链和企业集群，提高产业集中度，增强工业经济的整体竞争力。

　　提升现代服务业水平。依托我市产业和区位优势，制定全市第三产业发展规划，发展新型商贸业态。建设"第一楼街商业步行街"、"江鲜一条街"、九洲广场和农产品批发市场等重点工程。促进旅游业尽快成长为支柱产业。以打造滨江旅游带为重点，完成西津渡古街改造、润扬大桥桥区公园建设等重点旅游项目，启动世业洲旅游度假区开发，力争建成"三山"、茅山两个国家级风景名胜区。整合旅游资源，解决好发展旅游经济的整体配套问题。加大旅游宣传促销力度，加强与周边地区的联合，扩大旅游市场占有份额。以发展第三方物流为重点，强化物流基地建设，逐步把我市建设成为区域性物流中心。创新金融产品，加强银企合作，促进经济与金融的协调发展。

　　（三）突出沿江开发，争创对外开放新优势

　　加快基础设施建设。按照适度超前的原则，完善沿江规划，实施纵深开发。科学有序地开发、利用岸线和港口资源，吸引各种资本参与沿江开发。全力实施沪宁高速、312国道、镇溧高速、宁常高速、沿江高等级公路等重点新建和拓宽扩建项目。开工建设大港三期工程。加快公用码头、专用码头建设，建成万吨级泊位5个。加大开发区投资强度，完善基础设施配套，新增投入20亿元，增强集聚吸纳能力，形成以沿江开发为龙头的对外开放新格局。

　　增强招商引资实效。围绕镇江产业发展方向，延伸产业链招商，进一步扩大港资、台资规模，突出打造韩资高地、日资板块，挖掘欧美投资潜力。着力引进关联度大、牵引力强的战略性投资，引进超亿美元大项目和世界500强企业。抓住CEPA战略机遇，力争实现服务业利用港资的新突破。加强专业招商引资队伍建设，完善招商激励政策。创新招商理念，强化招商信息网络建设，务实推进规划招商、委托招商、网上招商、驻地招商、以商引商。完善以外资到位为主的考核机制。加快已批项目的建设步伐，年内建成投资在1000万美元以上的项目36个。

　　培植外贸外经亮点。主动适应国家出口退税机制改革，确保各项应对措施落到实处。重点培植一批规模型出口企业，大力发展民营进出口企业。充分发挥出口加工区的作用，吸引加工贸易企业落户镇江。巩固开发欧美日和东南亚等传统市场，积极拓展中东、俄罗斯、非洲、南美等新兴市场。扩大出口总量，优化产品结构，培育自有出口品牌，提高出口产品的技术含量和附加值。加快"走出去"步伐，鼓励大企业到境外投资。扩大对外服务贸易和技术贸易规模，开拓境外工程承包市场，提高劳务合作层次。

　　（四）突出体制创新，增强经济社会发展新动力

　　不断深化国企改革。加大攻坚力度，全面完成国有大中型骨干企业改革任务。加快破产企业终结进程，确保资产处置到位，人员分流落实到位。组织国企改革"回头看"，引导改制企业进一步完善机制，健全法人治理结构，加速资本扩张，实现企业的规范运作和健康发展。

　　大力发展民营经济。鼓励和引导非公有资本进入基础设施、公用事业等领域，参与国有、集体企业改制和资产重组。在税收政策、规费标准、市场准入、金融扶持、政府服务上，做到"五个平等"。帮助民营企业解决发展中的问题，完善中小企业融资担保体系，增设个体小额贷款金融品种。引导民营企业进一步加快体制创新和管理创新，使更多的民营企业走上现代企业之路。力争新增私营企业2800户、个体工商户1.3万户，私个经济增加值占GDP的比重提高5个百分点，吸纳民资100亿元。

　　整体推进各项改革。加快国有资产监督管理和运营体制改革，逐步实现政府公共管理职能和国有资产出资人职能分开。加快工业经济管理体制改革，培育一批适应市场经济要求、能够提供专业服务职能的行业协会。按照事权相统一的原则，调整市区财政体制。加快社会事业管理体制改革和事业单位转企改制，并在试点的基础上逐步推开。完善农村土地制度，依法保障农民对土地承包经营的各项权利。深化农村税费改革和粮食流通体制改革，农业税税率下调1个百分点，取消劳动积累工和义务工，探索对种粮农户的支持保护办法。

　　（五）突出特色个性，实现城市形象新提升

　　今年是"四年新提升"的开局之年，全口径城建总投资100亿元以上。以"打造南徐新城、凸现滨江特色、提升城市品位、建设生态镇江"为目标，精心营建独具个性、富有魅力的山水生态城市。坚持以规划指导建设，实施严格的规划控制。进一步拓宽城市经营思路，加快已注入土地入市交易步伐，积极探索各类城市资产市场化运作的新途径。

　　打造南徐新城。把南徐新城建设作为"四年新提升"的龙头工程，与国际权威规划机构合作，尽快制定一流的新城区规划。落实新行政中心选址和设计方案，启动南徐新城基础设施和公共设施建设，把南徐新城建成与南山风景区相辉映的花园式新城区，发挥带动辐射效应。

　　加快建设"生态镇江"。构筑西起世业洲、南接谷阳新城、东至江心洲的"城市绿色环廊"，年内完成总长12公里的西段。加快"水环境专项"实施步伐。完成5座山体的滑坡治理。启动搬迁并治理6家污染企业。努力提高国土绿化水平，人均公共绿地新增1平方米，建成国家园林城市。

　　完善城市综合功能。继续打造沿江、城东、城西三条发展主轴，进一步完善城市框架，完善城市主次干道和支路系统。完成跃进路、南徐西路、戴家门路、312国道主城段、经十二路南段、谷阳西南环、黄山北路等18条道路建设，总长46公里。加快天然气、电力、水利、通讯、环卫、人防等公用基础设施建设，建成市防震减灾指挥中心。新建3个城市公共停车场。加强社区基础设施建设，提升社区服务和管理水平，争创全国社区建设示范市。坚持长效管理，健全"统一领导、分级负责，以块为主、条块结合"的城市管理体制，扎实推进城市管理综合执法，巩固国家卫生城市和环保模范城市创建成果。

　　（六）突出协调发展，开创三个文明建设新局面

　　加强精神文明建设。扎实推进全国文明城市创建工作，塑造新时期镇江精神，激发广大干部群众热爱家乡、建设镇江的热情。倡导"爱国守法、明礼诚信、团结友善、勤俭自强、敬业奉献"的基本道德规范，深入开展"讲公民道德、做文明市民"教育活动。健全社会信用体系，打造"诚信镇江"，全面提高城市文明程度和市民道德水准。

　　推动各项社会事业全面发展。进一步提高九年义务教育和高中教育质量，深化素质教育，推进教育现代化建设，90%以上的乡镇基本达到省先进标准。加快市区中小学布局调整。支持在镇高校扩大规模，提高办学水平。加强职业教育，发展继续教育。高度重视人才的培训、引进和使用，完善人才市场服务体系。推进卫生事业的改革与发展，加强公共卫生应急体系建设，将各类城镇居民纳入医疗保障范围，实施特困职工医疗救助，扩大农村合作医疗大病保险的覆盖面。举办镇江市第十二届运动会，办好第十届全国运动会部分比赛项目。建设40个全民健身工程（点），有条件的广场全部设立"健身角"。积极推进文化体制改革，促进公益性文化事业和经营性文化产业的协调发展。提高人口与计划生育的服务水平和质量。深入开展"双拥"活动，做好人民武装工作。高度重视老龄工作。新建市救助管理站，救助和帮助特殊困难群众。做好统计、审计、民族宗教、外事、侨台、接待、机关事务管理、史志、档案、保密、气象等工作，继续发展妇女儿童、残疾人、关心下一代等社会事业。

　　推进民主法制建设。定期向人大及其常委会报告工作，自觉接受人民政协的民主监督，认真听取各民主党派、工商联、无党派代表人士和人民团体的意见。积极办理人大代表建议和政协提案。加强基层民主法制建设。大力推行政务、财务和社会公共事务公开，全面推进村民自治、社区自治。坚持从严治政、依法治市，深入开展"四五"普法，努力增强全社会的法律意识和法制观念。加强社会治安综合治理，严厉打击各类犯罪活动，创建"平安镇江"。进一步整顿和规范市场经济秩序，狠抓安全生产责任制的落实，防止重大案件或事故的发生。认真做好信访和人民调解工作，及时发现和化解不稳定因素，妥善处理新时期人民内部矛盾。努力维护社会稳定，为全市改革开放和经济建设创造良好的社会环境。

　　加强政府自身建设

　　做好改革、发展和稳定的工作，完成今年的各项任务，迫切需要我们围绕"创新、服务、高效、规范、清廉"的目标，建设现代政府。市政府各位组成人员和全体公务员都要进一步更新理念，加强学习，提高效能，用心、用智、用力把镇江的事情办好，对镇江人民负责。

　　我们将努力打造创新型政府。带着责任去创新。率先发展的实践，需要我们破除小胜即满、安于现状的思想；富民强市的重任，需要我们抛弃因循守旧、固步自封的陋习。勇于提出创新的举措，敢于承担创新的责任，把创新贯穿于政府工作的始终，努力用新的视角来审视我们的发展思路，用新的思维来分析我们面临的形势，用新的办法来解决我们前进中遇到的各种矛盾和问题。以与时俱进的精神状态、永续创新的更高要求，推动各项工作打开新局面。

　　我们将努力打造服务型政府。带着感情去服务。时刻牢记为人民服务是人民政府的宗旨，从群众最期盼的事情做起，切实把与群众生活密切相关的就业、保障、环保、住房、医疗和社会治安等问题紧紧抓在手上。在改革改制中更加注重职工安置，在征地拆迁中更加注重群众的合法权益，在工程建设中更加注重方便群众。深入到困难群众中去，深入到矛盾多的基层单位中去，真心实意地为群众排忧解难，尤其对弱势群体倾注更多的关爱，让千家万户有更多的笑容，有更多的温馨。

　　我们将努力打造高效型政府。以公开促高效。推广电子政务，办好"中国镇江"门户网站，形成统一的政务公开信息平台，突出公众服务网定位，拓宽政府与市民之间的沟通渠道，提高政府服务效能。倡导求真务实，大兴调研之风，切实精文简会，减少领导干部参加庆典和迎来送往，禁止观光旅游式的出国出访，不搞形式主义的达标评比活动，大力弘扬"说实话、干实事、求实效"的清新政风。加强行政效能监察，研究制定机关工作人员效能诫勉办法。

　　我们将努力打造规范型政府。以规范树形象。适应新的形势，加快政府职能转变。以《行政许可法》的贯彻实施为契机，进一步加强政府法制建设，提高依法行政的能力和水平。深化行政审批制度改革，清理各类政策文件，做到"应减必减"。完善市行政服务中心的运作机制，所有部门服务承诺、办事程序全部上墙，接受公众监督，最大程度便利投资者和人民群众。继续完善行政执法责任制、行政管理公示制、考核评议制和错案追究制，以硬手段治理软环境。

　　我们将努力打造清廉型政府。官正才能民安。按照"权力与责任挂钩，权力与利益脱钩"的要求，强化预算外资金的管理与审计，消除"公共权力部门化、部门权力利益化、部门利益私人化"现象，从源头上预防和遏制腐败。开展政府及各组成部门领导述廉评廉活动，强化制度约束，建立长效管理机制。充分发挥法律监督、民主监督、群众监督和舆论监督的作用，堵塞管理漏洞，降低行政成本，力戒奢侈浪费，政府带头过紧日子。

　　这里，我向全市人民承诺，建设"创新、服务、高效、规范、清廉"的现代政府，从我做起，从政府领导集体做起，从每一位政府组成人员做起。请各位代表、委员和全市人民监督！

　　各位代表，时代选择了我们，我们无上光荣；人民期待着我们，我们责无旁贷。让我们高举邓小平理论伟大旗帜，全面贯彻"三个代表"重要思想，在中共镇江市委的坚强领导下，团结和带领全市人民，坚定信心，扎实苦干，为率先全面建成小康社会、率先基本实现现代化而努力奋斗！
